# Modeling of Stochastic Wind Based on Operational Flight Data Using Karhunen–Loève Expansion Method

**DOI:** 10.3390/s20164634

**Published:** 2020-08-18

**Authors:** Xiaolong Wang, Lukas Beller, Claudia Czado, Florian Holzapfel

**Affiliations:** 1 Institute of Flight System Dynamics, Technical University of Munich, 85748 Garching, Germany; lukas.beller@tum.de (L.B.); florian.holzapfel@tum.de (F.H.); 2 Applied Mathematical Statistics, Technical University of Munich, 85748 Garching, Germany; cczado@ma.tum.de

**Keywords:** wind model, operational flight data, stochastic process, Karhunen–Loève Expansion, vine copula, spectral representation

## Abstract

Wind has a significant influence on the operational flight safety. To quantify the influence of the wind characteristics, a wind series generator is required in simulations. This paper presents a method to model the stochastic wind based on operational flight data using the Karhunen–Loève expansion. The proposed wind model allows us to generate new realizations of wind series, which follow the original statistical characteristics. To improve the accuracy of this wind model, a vine copula is used in this paper to capture the high dimensional dependence among the random variables in the expansions. Besides, the proposed stochastic model based on the Karhunen–Loève expansion is compared with the well-known von Karman turbulence model based on the spectral representation in this paper. Modeling results of turbulence data validate that the Karhunen–Loève expansion and the spectral representation coincide in the stationary process. Furthermore, construction results of the non-stationary wind process from operational flights show that the generated wind series have a good match in the statistical characteristics with the raw data. The proposed stochastic wind model allows us to integrate the new wind series into the Monte Carlo Simulation for quantitative assessments.

## 1. Introduction

To improve the operational flight safety, an Acceptable Level of Safety Performance (ALoSP) [1] is defined to identify the safety level for airlines or airports. Wind has a significant influence on operational flight safety and should be considered in the quantitative assessments of the operational flight safety level. Mathematical wind models such as ’1-cosine’ discrete wind gust model, low-level wind shear model, von Karman turbulence model, and Dryden turbulence model, are commonly used for flight simulation and flight control design [2,3,4]. European Aviation Safety Agency (EASA) also illustrated the models of mean wind, turbulence, and wind shear for airworthiness assessment [5]. However, the general wind models cannot represent the local statistical characteristics of the wind. Therefore, they are not suitable to quantify the operational risk for a specific airline or airport. Instead, wind estimated from operational flight data in the onboard Quick Access Recorder (QAR) [6,7], as a sufficient wind database, allows us to analyze the specific statistical characteristics of the real wind conditions.

A model-based predictive analysis framework to quantify the incident probability for a specific airline has been developed by the Institute of Flight System Dynamics [8,9]. Wind speed, as one of the contributing factors to the incident, can be taken into account in this approach. The quantitative assessment of the incident probability is obtained via propagating the uncertainty of the contributing factors through the incident model. During the uncertainty propagation, each contributing factor remains constant in one evaluation of the incident model. As shown in the runway overrun case in the paper [8], the wind speed is constant in the incident model. However, the constant wind assumption is not suitable for all incidents, as the variation of wind speed and direction affects the flight performance and handling quality significantly. Therefore, wind series should be considered in the incident model. Although the traditional wind shear or turbulence model can be used in Monte Carlo simulation, they can not represent the statistical characteristics of the specific operational flight data. Thus, the objective of this paper is to build a stochastic wind model in order to generate new wind series, which follow the statistical characteristics of the real wind data and can be used in the incident model simulation.

Wind series can be assumed as a stochastic process. Conventional turbulence wind is modeled as a stationary stochastic process using the spectral representation (SR) methods [10]. However, such approaches can not deal with the non-stationary wind series. To obtain the realistic quantitative statement of the operational flight risk, a high fidelity model to describe the wind series is required. Markov processes are highly useful models for stochastic processes, but the process has to satisfy the Markov property [11]. Without any assumption about the process, a Karhunen–Loève (KL) expansion method is proposed in [12,13] to construct the observed non-stationary random phenomena, such as seismic ground motion and wave field. This method is also called functional principal component analysis [14], which are widely used in sensitivity analysis domain to identify the key parameters and also has a good performance in dimension reduction. In addition, some researchers show that the SR method and the KL expansion method coincide for the weakly stationary process [15,16]. In this paper, we apply the KL expansion method to model the encountered wind series during the operational flights. The coefficients in KL expansion are assumed to be independent in many applications [14]. However, it is not always true, especially if the stochastic process is not Gaussian process [12]. The pair dependence between every two coefficients in KL is considered in [13]. To better describe the statistical characteristics of the KL coefficients, a vine copula technique [17] is used to capture the high dimensional dependence among those coefficients instead of the pair dependence. With the integration of a vine copula into KL expansion, the better representation of KL coefficients would improve the accuracy of the constructed stochastic wind model. To compare with the conventional turbulence model used in aviation, the similarity and difference between the KL and the SR method for turbulence modeling are also discussed in this paper.

The paper starts with an explanation about the basic concepts of the KL expansion method and vine copula, followed by a comparison of SR and KL in terms of equations in Section 2. In Section 3, the modeling results of turbulence data and real headwind series data are presented and discussed. Besides, the modeling of extracted wind shear ramps is also implemented. Section 4 contains conclusions and possible improvements of the proposed wind construction model.

## 2. Modeling of Stochastic Process

Two main construction methods of the stochastic process are discussed in this section: KL expansion and SR methods. The KL expansion method is introduced to model the finite stochastic process. Then, a vine copula is brought in to construct the high dimensional dependence of the random variables in the KL expansion. The strategy that the vine copula describes the high dimensional dependence is explained using a three-dimensional case. Besides, we present the SR method for the stationary process, which is subsequently compared with the KL expansion.

### 2.1. Karhunen–LoèVe Expansion

Let X(t) be a stochastic process at a given time interval t∈[0,T]. The autocorrelation and autocovariance functions are usually used to describe the random process. They are calculated as follows:

(1) $$\begin{array}{cc} R(t_{1},t_{2}) & =E[X\left(t_{1}\right)X\left(t_{2}\right)],\\ Cov(t_{1},t_{2}) & =E\left[\left(X\left(t_{1}\right)-\mu_{1}\right)\left(X\left(t_{2}\right)-\mu_{2}\right)\right]=R\left(t_{1},t_{2}\right)-\mu_{1}\mu_{2}, \end{array}$$

where $t_{1},t_{2}\in T$. E is the expectation operator, and $\mu_{i}$ is the mean of the process X(t) at given time $t_{i}$. For a zero-mean stochastic process, the autocorrelation and autocovariance are equivalent. As an arbitrary process can be transformed to a zero-mean process by subtracting its mean, without loss of generality, we can assume that X(t) is a zero-mean process. Then, X(t) can be reconstructed using the KL expansions [18]:

(2) $$X\left(t\right)=\sum_{k=1}^{\infty }\sqrt{\lambda_{k}}\xi_{k}\phi_{k}\left(t\right),$$

where the $\lambda_{k}$ and the corresponding functions $\phi_{k}\left(t\right)$ are the solutions of the integral equation:

(3) $$\int_{D}R\left(t,s\right)\phi \left(s\right)ds=\lambda \phi \left(s\right),\;\;t,s\in D=\left[0,T\right].$$

The corresponding random coefficients $\xi_{k},k=\left\{1,2,\ldots \right\}$ are computed using

(4) $$\xi_{k}=\frac{1}{\sqrt{\lambda_{k}}}\int_{D}X\left(t\right)\phi_{k}\left(t\right)dt.$$

By Mercer’s theorem [18], the R(t,s) can be expressed in terms of its eigenvalues and eigenvectors via eigendecomposition as follows:

(5) $$R\left(t,s\right)=\sum_{k=1}^{\infty }\lambda_{k}\phi_{k}\left(t\right)\phi_{k}\left(s\right),$$

where the obtained $\lambda_{k}$ and $\phi_{k}\left(t\right)$ fulfill Equation (3). In addition, eigenvectors $\phi_{k}\left(t\right)$ are orthonormal and their inner product follows the equation:

(6) $$<\phi_{k}\left(t\right),\phi_{l}\left(t\right)>=\int_{D}\phi_{k}\left(t\right)\phi_{l}\left(t\right)dt=\delta_{kl},\;\;\;k,l\geq 1,$$

where $\delta_{kl}$ is the Kronecker delta function. In addition, X(t) is a zero-mean process, the total variance of the X(t) can be derived as below:

(7) $$\begin{array}{ccc} \int_{D}Var\left[X\left(t\right)\right]dt & =\int_{D}E\left[X^{2}\left(t\right)\right]dt\; & =\int_{D}E\left[{\left(\sum_{k=1}^{\infty }\sqrt{\lambda_{k}}\xi_{k}\phi_{k}\left(t\right)\right)}^{2}\right]dt=\sum_{k=1}^{\infty }\lambda_{k}. \end{array}$$

The sum of all $\lambda_{k}$ indicates the integrated variance of the process. In practice, Equation (2) is truncated at a certain order *K*. Therefore, the relative error ϵ of the truncated expansion can be calculated as below:

(8) $$\epsilon =1-\frac{\sum_{k=1}^{K}\lambda_{k}}{\int_{D}R\left(t,t\right)dt}=1-\frac{\sum_{k=1}^{K}\lambda_{k}}{\sum_{k=1}^{\infty }\lambda_{k}}.$$

1−ϵ is called the cumulative variance ratio. As mentioned before, X(t) is a process with zero mean. It means that E[X(t)]=0 for all t∈D. Considering an arbitrary process Y(t), the truncated KL expansion series of an arbitrary process can be represented by adding its mean as below:

(9) $$Y\left(t\right)\approx E\left[Y\left(t\right)\right]+\sum_{k=1}^{K}\sqrt{\lambda_{k}}\xi_{k}\phi_{k}\left(t\right),$$

where $\lambda_{k}$ is sorted in descending order as $\lambda_{1}\geq \lambda_{2}\geq \;\ldots \;\geq 0$. As the eigenvectors $\phi_{k}$ are orthonormal and Y(t)−E[Y(t)] is zero mean, the realizations of $\xi_{k}$ computed using Equation (4) satisfy

(10) $$E\left[\xi_{k}\right]=0,\;Var\left[\xi_{k}\right]=1,\;E\left[\xi_{k}\xi_{l}\right]=\delta_{kl},$$

which indicates that random variables $\xi_{k}$ are mutually uncorrelated to each other, and distributed with zero mean and unit variance. In a particular case that the random process is a Gaussian process, all $\xi_{k}$ are independent standard Gaussian distributed. For the non-Gaussian process, the dependence among $\xi_{k}$ can be described by the joint distribution of $\xi_{k}$. New samples of $\xi_{k}$ sampled from the distribution of $\xi_{k}$ allow us to generate new realizations of the stochastic process using Equation (9).

### 2.2. Vine Copula

The integration of a vine copula into KL expansion is proposed to deal with the dependent $\xi_{k}$. A brief introduction related to the copula is presented. For details, the readers are referred to [17,19]. Based on the theorem of Sklar [20], the joint distribution *F* of the dependent $\xi_{k}$ can be decomposed by the marginal cumulative distribution functions (CDF) $F_{k}$ and a copula structure *C* as below:

(11) $$F\left(\xi_{1},\xi_{2},\ldots ,\xi_{K}\right)=C\left(F_{1}\left(\xi_{1}\right),F_{2}\left(\xi_{2}\right),\ldots ,F_{K}\left(\xi_{K}\right)\right).$$

As $F_{k}$ is the CDF of $\xi_{k}$, the realization of $F_{k}$ is uniformly distributed. *C* describes the dependence among $\xi_{k}$ and eliminates the impact of the marginal distributions. The probability density *f* is computed as below:

(12) $$f\left(\xi_{1},\xi_{2},\ldots ,\xi_{K}\right)=c\left(F_{1}\left(\xi_{1}\right),F_{2}\left(\xi_{2}\right),\ldots ,F_{K}\left(\xi_{K}\right)\right)\prod_{k=1}^{K}f_{k}\left(\xi_{k}\right).$$

As the truncated order *K* of the KL expansion might go to 20 or more for highly accurate reconstruction, the copula density function *c* becomes too complex. Based on the Rosenblatt transformation [21] and the conditioning arguments [17], the vine copula approach allows us to construct a *K*-dimensional copula density function using $\frac{K(K-1)}{2}$ bivariate copula densities. The maximum likelihood method is applied to estimate the bivariate copula densities based on the given data. An example of a three-dimensional probability distribution constructed from three bivariate copula densities is presented in Appendix A. Since the construction of high dimensional copula in Equation (A3) is not unique, [22] describes a sequential strategy to obtain the most suitable construction structure. After the vine copula dependence model of $\xi_{k}$ is obtained, dependent samples of the $\xi_{k}$ can be generated using the vine copula. Based on the KL expansion and vine copula methods, the procedure of the stochastic process reconstruction and regeneration is summarized in Algorithm 1.

**Algorithm 1** Procedure of the stochastic process reconstruction and regeneration
1:Collection of the measured realizations of the stochastic process *Y*2:Center the data *Y* using X(t)=Y(t)−E[Y(t)]3:Calculate the autocovariance R(t,s) of *X* using Equation (1)4:Obtain the eigenfunctions $\phi_{i}$ and eigenvalues $\lambda_{i}$ via the eigendecomposition of R(t,s) using Equation (5)5:Calculate the cumulative variance ratio 1−ϵ using Equation (8) and set the truncated order *K* depending on the required 1−ϵ.6:Calculate the coefficients $\xi_{k}$, where k=1,2…,K using Equation (4)7:**if** Reconstruct the original process **then**8:    Calculate the $Y_{rec}$ using the computed $\xi_{k}$ based on the Equation (9)9:
**end if**
10:**if** Regenerate the new process **then**11:    Estimate the marginal distributions of $\xi_{k}$12:    **if** Independent Sampling **then**13:        Get samples of $\xi_{k}$ via sampling individually from the marginal distributions14:    **else if** Copula Sampling **then**15:        Estimate the vine copula of $\xi_{k}$.16:        Get samples of $\xi_{k}$ via the copula sampling17:    **end if**18:    Calculate the $Y_{new}$ using the sampled $\xi_{k}$ based on the Equation (9)19:
**end if**



### 2.3. Spectral Representation

The SR method is often applied to deal with a stationary stochastic process. SR describes the features of the process in the frequency domain using the power spectral density (PSD) function P(ω). P(ω) can be computed via the Fourier transformation of the autocovariance function $R_{X}\left(t+\tau ,t\right)$ of the process [23] as below:

(13) $$P\left(\omega \right)=\int_{-\infty }^{\infty }R_{X}\left(t+\tau ,t\right)e^{-i\omega \tau }d\tau ,$$

where ω is the angular frequency and τ is the time lag between two time points. $R_{X}\left(t+\tau ,t\right)$ = $R_{X}\left(\tau \right)$ for the stationary process, and it can be obtained based on the inverse Fourier transformation as below:

(14) $$R_{X}\left(t+\tau ,t\right)=\frac{1}{2\pi }\int_{-\infty }^{\infty }P\left(\omega \right)e^{i\omega \tau }d\omega .$$

For a finite stationary stochastic process with zero-mean X(t),t∈[0,T], it can be represented using the exponential form of the Fourier series expansion as below:

(15) $$X\left(t\right)=\sum_{k=-\infty }^{\infty }F_{k}e^{ik\Delta \omega t}=\sum_{k=-\infty }^{\infty }\left|F_{k}\right|e^{i\psi_{k}}e^{i\omega_{k}t},$$

where $\omega_{k}=k\Delta \omega =k\cdot 2\pi /T$ and $F_{k}$ is the Fourier coefficients. $|F_{k}|$ and $\psi_{k}$ are the frequency amplitude and corresponding phase, respectively. $F_{k}$ can be calculated via the Fourier inverse transformation as below:

(16) $$F_{k}=\frac{1}{T}\int_{0}^{T}X\left(t\right)e^{-i\omega_{k}t}dt.$$

The average power of the process *P* is defined as

(17) $$P=\frac{1}{T}\int_{0}^{T}X^{2}\left(t\right)dt=\frac{1}{T}\int_{0}^{T}{\left(\sum_{k=-\infty }^{\infty }F_{k}e^{i\omega_{k}t}\right)}^{2}dt.$$

Since the inner product of the exponential terms in Equation (eq:aveP) satisfies

(18) $$<\frac{e^{i\omega_{k}t}}{\sqrt{T}},\frac{e^{i\omega_{l}t}}{\sqrt{T}}>=\int_{0}^{T}\frac{e^{i\omega_{k}t}}{\sqrt{T}}\frac{e^{-i\omega_{l}t}}{\sqrt{T}}dt=\delta_{kl},$$

the average power *P* can be rewritten as below:

(19) $$P=\sum_{k=-\infty }^{\infty }{\left|F_{k}\right|}^{2}=\sum_{k=-\infty }^{\infty }P_{k},$$

where $P_{k}$ is the power for the specific $\omega_{k}$. Substitute $|F_{k}|$ using $\sqrt{P_{k}}$ in Equation (15):

(20) $$X\left(t\right)=\sum_{k=-\infty }^{\infty }\sqrt{P_{k}}e^{i\psi_{k}}e^{i\omega_{k}t}=\sum_{k=-\infty }^{\infty }\sqrt{P_{k}T}e^{i\psi_{k}}\frac{e^{i\omega_{k}t}}{\sqrt{T}}.$$

Therefore, $R_{X}\left(\tau \right)$ in Equation (14) can be rewritten in the Fourier series expansion:

(21) $$R_{X}\left(\tau \right)=\sum_{k=-\infty }^{\infty }P_{k}e^{i\omega_{k}\tau }=\sum_{k=-\infty }^{\infty }P_{k}e^{i\omega_{k}\left(t-s\right)}=\sum_{k=-\infty }^{\infty }P_{k}T<\frac{e^{i\omega_{k}t}}{\sqrt{T}},\frac{e^{i\omega_{k}s}}{\sqrt{T}}>,$$

where t,s∈T. For an infinite stationary process, P(ω) can be obtained by taking the limit of $P_{k}T$ when T→∞ as follows:

(22) $$P\left(\omega \right)=\lim_{\Delta \omega_{k}\to 0}\frac{2\pi P_{k}}{\Delta \omega_{k}}=\lim_{T\to \infty }P_{k}T,\;for\;\omega_{k}\in \left[\omega_{k-1},\omega_{k}\right],$$

where $\Delta \omega_{k}=\omega_{k}-\omega_{k-1}$. Compare the representation equation Equation (15) with the KL expansion equation Equation (2), and the autocovariance equation Equation (21) with Equation (5), respectively. It shows that $\lambda_{k}=P_{k}T$ and $\phi_{k}=e^{i\omega_{k}t}/\sqrt{T}$ are the solution of the integral equation Equation (3). It reveals that the SR and the KL expansion coincide in the construction of a finite stationary process. For the infinite stationary process, it is worth mentioning that P(ω) is continuous and has two sides ω<0 and ω>0. However, the $\lambda_{k}$ in KL expansion method is discrete and the corresponding $\omega_{k}$ is always larger than 0.

## 3. Results and Discussion of Wind Process Modeling

This section starts with a turbulence model construction. Wind data generated from a well-known von Karman turbulence model is used to compare the KL expansion and the SR method. Then, the wind series during the operational flight is reconstructed and regenerated using the KL expansion method. The original headwind data is estimated based on 849 flights of a B747-8F that landed on the same runway. A preprocessing of the data is implemented using the Rauch-Tung-Striebel smoother smoother to improve the data quality. For the details of the data smoothing, readers are referred to reference given in [24]. Modeling of headwind data during the final approach is presented, followed by another case study of the horizontal wind shear ramps construction.

### 3.1. Turbulence

The conventional wind turbulence model in aviation is described using the PSD function of the SR method. For an aircraft flying at speed *V* through a frozen turbulence field, PSD functions Φ(Ω) of the von Karman model [10] in three directions are shown as below:

(23) $$\begin{array}{ccc} \Phi_{u}\left(\Omega \right) & =\sigma_{u}^{2}\frac{2L_{u}}{\pi }\frac{1}{{\left[1+{\left(1.339L_{u}\Omega \right)}^{2}\right]}^{5/6}}, & \left(Longitudinal\right)\\ \Phi_{v}\left(\Omega \right) & =\sigma_{v}^{2}\frac{L_{v}}{\pi }\frac{1+\frac{8}{3}{\left(1.339L_{v}\Omega \right)}^{2}}{{\left[1+{\left(1.339L_{v}\Omega \right)}^{2}\right]}^{11/6}}, & \left(Lateral\right)\\ \Phi_{w}\left(\Omega \right) & =\sigma_{w}^{2}\frac{L_{w}}{\pi }\frac{1+\frac{8}{3}{\left(1.339L_{w}\Omega \right)}^{2}}{{\left[1+{\left(1.339L_{w}\Omega \right)}^{2}\right]}^{11/6}}, & \left(Vertical\right) \end{array}$$

where *u*, *v* and *w* refer to the longitudinal, lateral and vertical turbulence, respectively. $\sigma_{u}$, $\sigma_{v}$, and $\sigma_{w}$ are the intensity measures of the turbulence. $L_{u}$, $L_{v}$, and $L_{w}$ (in feet) are the length scales of the turbulence. Ω is the spatial frequency, which is equal to the angular frequency ω divided by *V*. In general, ω is in the range of [0,100π] [10]. With respect to ω, the PSD S(ω) is computed as follows:

(24) $$S\left(\omega \right)=\frac{\Phi (\Omega )}{V}=\frac{\Phi (\omega /V)}{V}.$$

**Figure 1:**
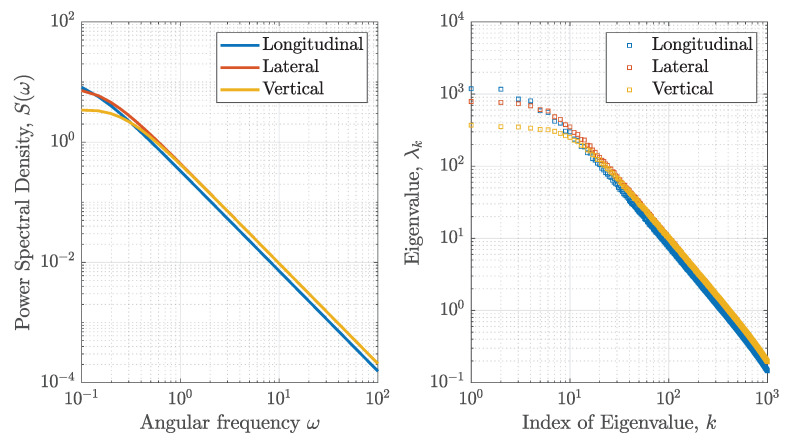
Comparison of the SR and KL construction for turbulence data in a certain flight condition.

**Figure 2:**
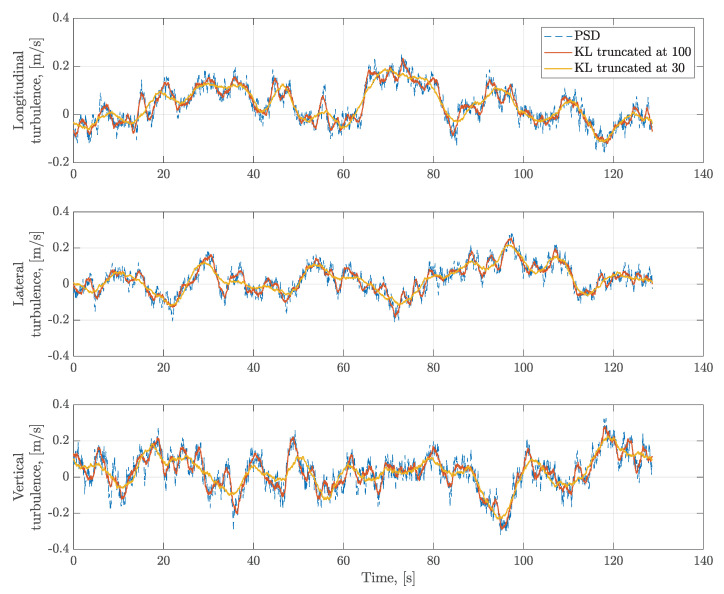
Comparison between the generated turbulence based on the PSD and the reconstructed turbulence using the KL expansion.

When the altitude of aircraft *h* (in feet) is less than 1000 feet, the *L* and σ can be calculated as below:

(25) $$\begin{array}{cc} L_{w} & =h,\\ L_{u} & =L_{v}=\frac{h}{{\left(0.177+0.000823h\right)}^{1.2}}, \end{array}$$

(26) $$\begin{array}{cc} \sigma_{w} & =0.1V_{W20},\\ \sigma_{u} & =\sigma_{v}=\frac{\sigma_{w}}{{\left(0.177+0.000823h\right)}^{0.4}}, \end{array}$$

where $V_{W20}$ is the wind speed measured at 20 feet (6.10 m). Given a certain flight condition, for example, *h* = 600 feet (182.88 m), $V_{W20}$ = 15 knots (7.72 m/s), and *V* = 140 knots (72.02 m/s), the PSD of the turbulence model in three directions is shown in Figure 1. 2000 time series of 256 s in 16 Hz are generated for each direction using the Equation (23) in the same flight condition. One example of the generated time series is shown using the blue dash line in Figure 2. The KL expansion method is implemented based on the generated 2000 turbulence series. Compared with the PSD, eigenvalues with the corresponding index in KL expansion are also shown in Figure 1.

Figure 1 shows that the shape of PSD and eigenvalues in the KL expansion are similar. As mentioned in Section 2.3, the index *k* of the KL expansion terms corresponds to a certain angular frequency $\omega_{k}$ and the eigenvalue $\lambda_{k}$ in the KL expansion is equivalent to the PSD P(ω). The PSD of the turbulence model decreases with an increase of ω. Meanwhile, the eigenvalues $\lambda_{k}$ in the KL expansion are sorted. Thereby, the shape of PSD and eigenvalues is almost similar. This result validates that the KL expansion method coincides with the SR method for the stationary stochastic process.

**Figure 3:**
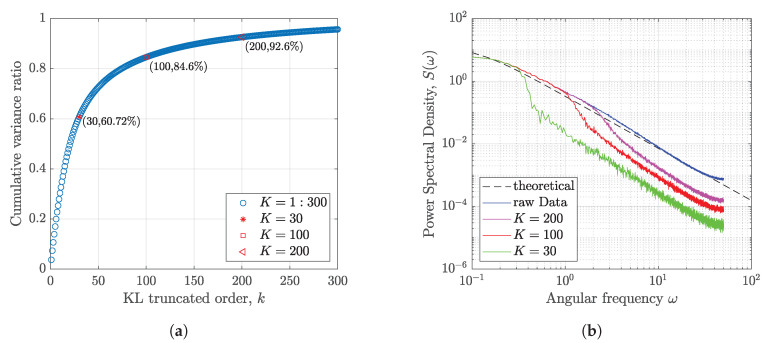
KL expansion results of longitudinal turbulence. (**a**) Cumulative variance ratio of KL expansion; (**b**) PSD comparison of reconstructed turbulence.

Two truncated orders K=30 and K=100 are selected in the KL expansion of turbulence. One example of KL reconstructed results is shown in Figure 2. It shows that the reconstructed process and the series generated from the PSD match very well. The reconstructed accuracy is improved with the increase of the truncated order. As the high order term is corresponding to the high frequency in this case, higher frequencies are neglected in the lower order truncated KL reconstruction. To quantify the influence of the KL truncated order *K*, the cumulative variance ratio 1−ϵ of the KL expansions in the case of longitudinal turbulence is calculated using Equation (8) and also shown in Figure 3a. It shows that the reconstructed turbulence with K=100 can achieve 84.6% of variance. When *K* increases to 200, 92.6% of variance is achieved. Furthermore, the PSDs of the 2000 reconstructed longitudinal turbulence series using KL expansions are computed and shown in Figure 3b. The ’theoretical’ black line is computed directly based on the von Karman model using Equation (23). The ’raw data’ indicates the PSD are cumputed based on the turbulence data generated from the von Karman model. The ’*K* = 200, 100, 30’ shows the mean of PSD computed based on the KL reconstructed turbulence data with difference truncated orders. It also shows that the accuracy of PSD obtained from reconstructed data will be improved with the increasing order *K*. This property is also indicated by the cumulative variance ratio. Nevertheless, the KL expansion with sufficient orders based on the turbulence data is consistent with the conventional von Karman model. This approach allows us to construct the turbulence wind process.

### 3.2. Headwind

**Figure 4:**
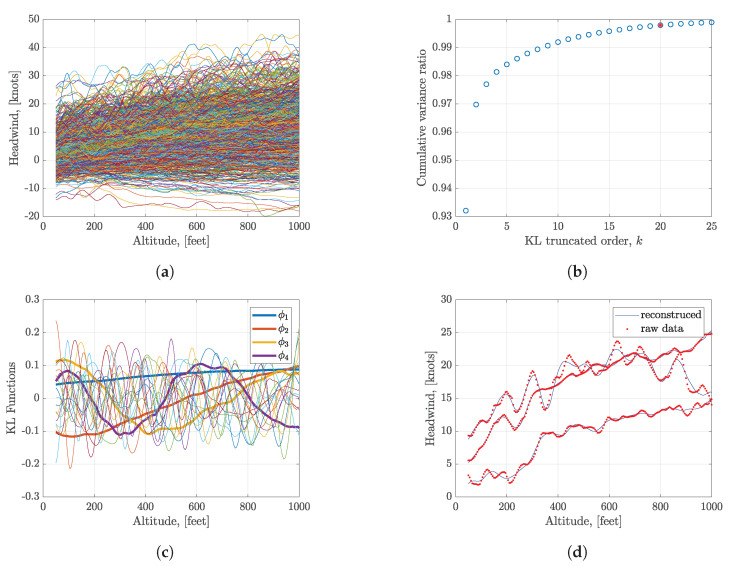
Headwind series and KL expansion results. (**a**) Headwind of 849 flights; (**b**) Cumulative variance ratio of KL expansion; (**c**) Eigenfunctions of KL expansion; (**d**) Reconstruction of three arbitrary wind series.

Headwind has a significant influence on landing performance. A case of headwind process construction is demonstrated in this section. The headwind from 1000 feet (304.80 m) to 50 feet (15.24 m) above ground level (AGL) is estimated from the operational flight data in the final approach phase. 849 series of headwind (in knots) are resampled from the smoothed headwind based on the altitude (in feet) shown in Figure 4a. All wind series are homogenized after resampling.

The KL expansion method is implemented based on the headwind series of 849 flights. Figure 4b shows the relationship between the cumulative variance ratio and the truncated order *K*. K=20 is selected in this case, and more than 99.9% variance of the process is obtained. Eigenfunctions $\phi_{k}$ of the autocorrelation matrix are shown in Figure 4c. According to the Equation (9), three arbitrary reconstructed wind series using the calculated $\xi_{k}$ are illustrated and compared with raw wind series data in Figure 4d. Although only 20 terms KL functions are used in the construction, the reconstructed wind series and raw wind series match very well, which indicates that truncated KL expansion can be used to reconstruct the headwind series.

**Figure 5:**
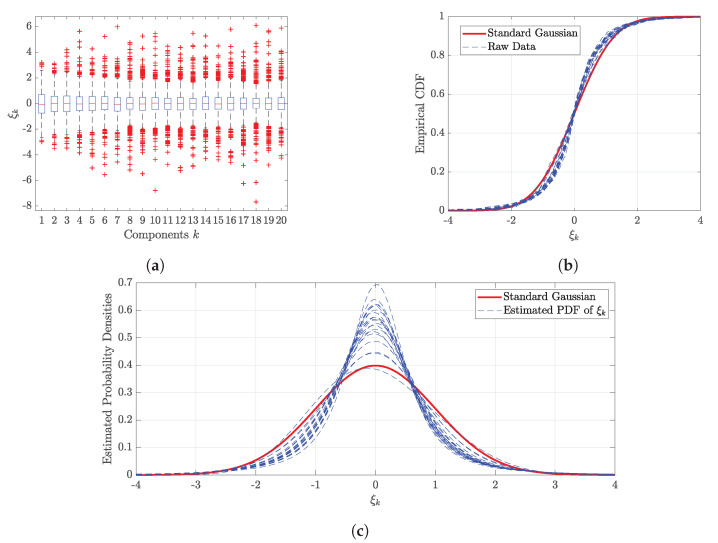
Marginal distributions of $\xi_{k}$. (**a**) Box plot of $\xi_{k}$; (**b**) Empirical CDF of $\xi_{k}$; (**c**) Estimated PDF of $\xi_{k}$.

New samples of the $\xi_{k}$ are required to generate the new wind series. The marginal distribution of each $\xi_{k}$ is studied. Box plot of $\xi_{k}$ in Figure 5a shows the computed $\xi_{k}$ are distributed close to zero mean and unit variance, which is also derived in Equation (10). However, Figure 5a also reveals that the median of $\xi_{k}$ is not always zero. The empirical CDF of $\xi_{k}$ and standard Gaussian distribution are compared in Figure 5b. All blue dash lines are related to the empirical CDF of $\xi_{k}$. It indicates that some of $\xi_{k}$ are almost following the standard Gaussian distribution. However, some deviations of CDF do exist in this case. Furthermore, marginal distributions for all $\xi_{k}$ are estimated using maximum likelihood methods from the distribution candidates, such as ’Gaussian’, ’Student-t’, ’Generalized Extreme Value’, ’t Location-Scale’, ’Logistic’ distributions, and so on. Estimated probability densities of $\xi_{k}$ are compared in Figure 5c, and the corresponding distribution parameters are shown in Table A1 of Appendix B. Deviations in PDF between the estimated distributions and Gaussian distribution are obvious. Instead of Gaussian distribution, the estimated marginal distributions of $\xi_{k}$ are used to generate the realizations of $\xi_{k}$, which are subsequently used for the new wind series production based on the Equation (9).

**Figure 6:**
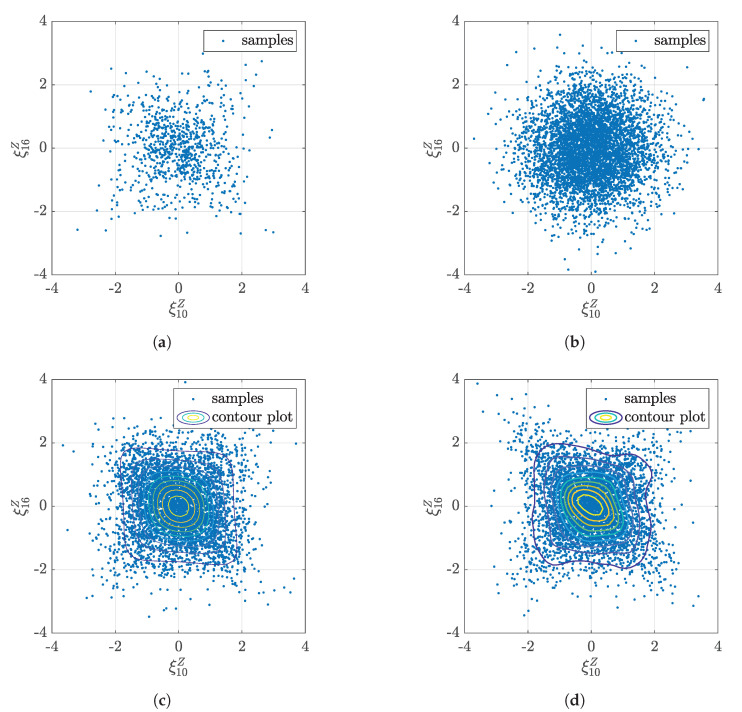
Different sample sets of $\xi_{10}$ and $\xi_{16}$ in *Z* scale (standard Gaussian space). (**a**) Raw samples; (**b**) Independent sampling; (**c**) Parametric copula sampling; (**d**) Nonparametric copula sampling.

**Figure A1:**
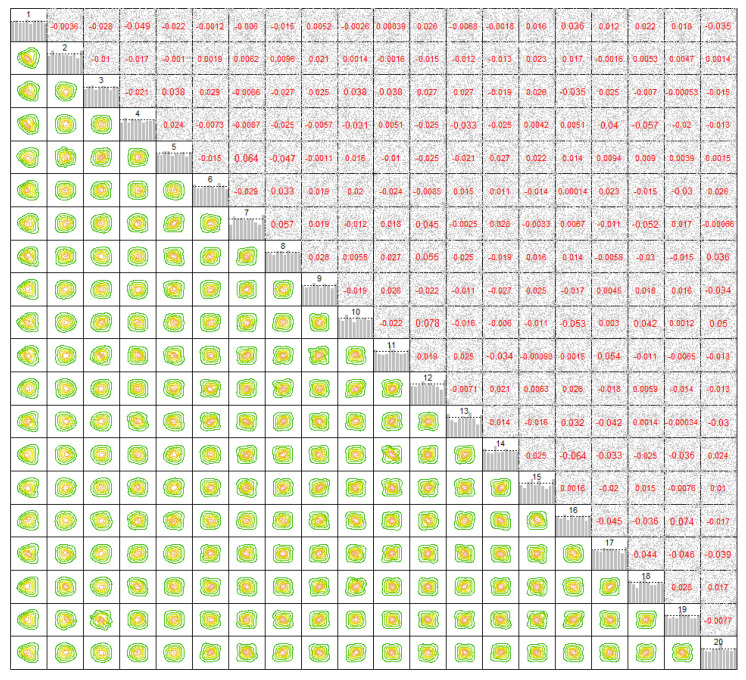
Empirical normalized pairwise contour plots in the headwind modeling (generated using R package “VineCopula” [25]).

**Figure A2:**
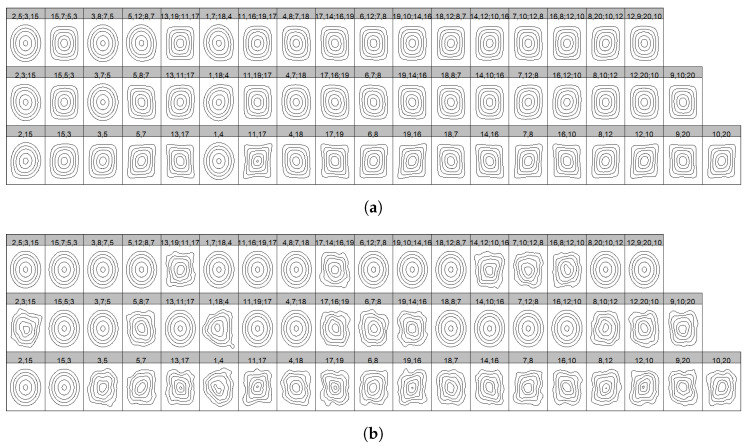
Normalized contour plots of bivariate copulas in headwind modeling ( generated using R package ’rvinecopulib’ [26]). (**a**) Contour plots using the parametric bivariate copula; (**b**) Contour plots using the nonparametric bivariate copula.

As mentioned before, dependence among $\xi_{k}$ might exist for non-Gaussian distributed $\xi_{k}$. A vine copula approach is applied to capture the dependence of the obtained $\xi_{k}$. To analyze the pairwise dependence between two $\xi_{k}$, the empirical contour plots using the copula data are shown in Figure A1 of Appendix C. The copula data has three scales: *X* scale denoted by $\xi_{k}^{X}$, *U* scale denoted by $\xi_{k}^{U}$, and *Z* scale denoted by $\xi_{k}^{Z}$. $\xi_{k}^{X}$ is the same with the raw data $\xi_{k}$. $\xi_{k}^{U}$ is obtained via transforming all samples of $\xi_{k}$ into the uniform space as follows:

(27) $$\xi_{k}^{U}=F_{k}\left(\xi_{k}\right),$$

where $F_{k}$ is the CDF of the marginal distribution of $\xi_{k}$. $\xi_{k}^{Z}$ is used in the normalized contour plots, and it is obtained via transforming all samples of $\xi_{k}$ into the standard Gaussian space using:

(28) $$\xi_{k}^{Z}=\Psi^{-1}\left(F_{k}\left(\xi_{k}\right)\right),$$

where Ψ is the CDF of the standard Gaussian distribution. Figure A1 shows that most dependence between two $\xi_{k}$ is small, but some pair dependence exist, for example, the dependence between $\xi_{10}$ and $\xi_{16}$. A vine copula with the estimated marginal distributions is used in this case to capture the dependence. The parametric and nonparametric bivariate copula are used separately and normalized contour plots on the Z scale are shown in Figure A2a,b, respectively. The parametric bivariate copula is using the defined copula function to fit the data, and the unknown parameters in the copula functions are estimated [17]. In contrast, the nonparametric bivariate copula is using the empirical function for two variables, which is the same as the empirical CDF for one variable. New realizations of $\xi_{k}$ can be generated via independent sampling, parametric vine copula sampling, and nonparametric vine copula sampling. To visualize the difference of generated samples, sample plots between $\xi_{10}$ and $\xi_{16}$ are shown in Figure 6. The raw data (Figure 6a), independent samples (Figure 6b), parametric (Figure 6c) and nonparametric copula samples (Figure 6d) in *Z* scale are illustrated, respectively. The dependence between $\xi_{10}$ and $\xi_{16}$ is evident in this case. Compared with the individually sampling from the marginal distributions, samples generated using the vine copula capture the dependence of the raw data and thus give a better representation of the raw data.

**Figure 7:**
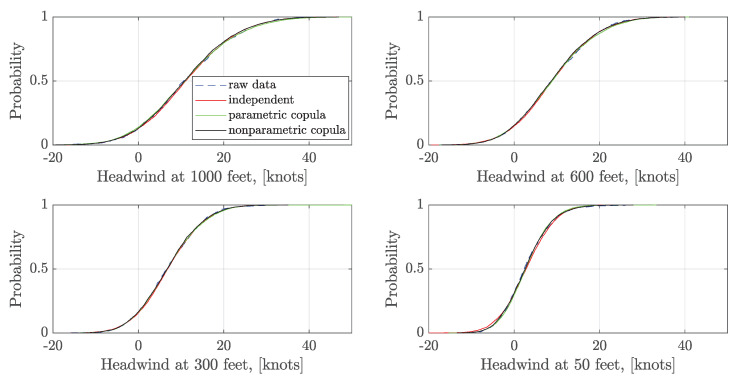
Comparison of the empirical CDFs of headwind at a certain altitude.

**Figure 8:**
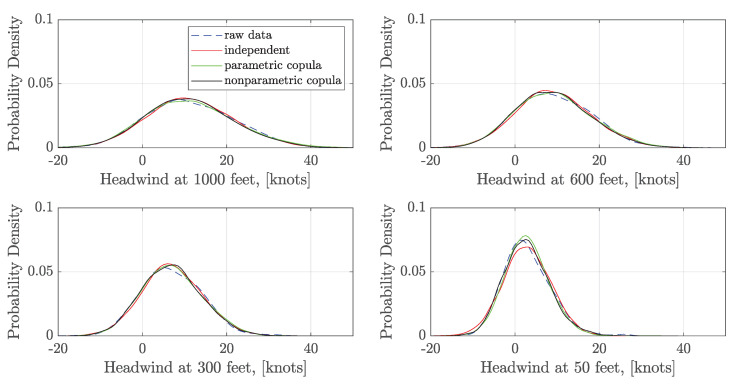
Comparison of the estimated PDFs of headwind at a certain altitude.

The statistical characteristics of the generated wind series are analyzed to validate the construction results. 5000 samples of $\xi_{k}$ are generated using the independent sampling and the vine copula sampling. Then the corresponding 5000 wind series are generated for independent samples and dependent samples, respectively. The empirical CDFs and the kernel fitted PDFs of headwind speed at four different altitudes: 1000 feet (304.80 m), 600 feet (182.88 m), 300 feet (91.44 m), and 50 feet (15.24 m) are shown in Figure 7 and Figure 8. It indicates that the generated wind series obtain a similar statistical distribution at a certain altitude compared with the raw data. Moreover, there is no significant difference in the headwind distribution between the independent and dependent sampling in 1000 feet, 600 feet, 300 feet. However, headwind distribution densities at 50 feet using the parametric and nonparametric copula sampling are closer to the raw data than the independent sampling.

**Figure 9:**
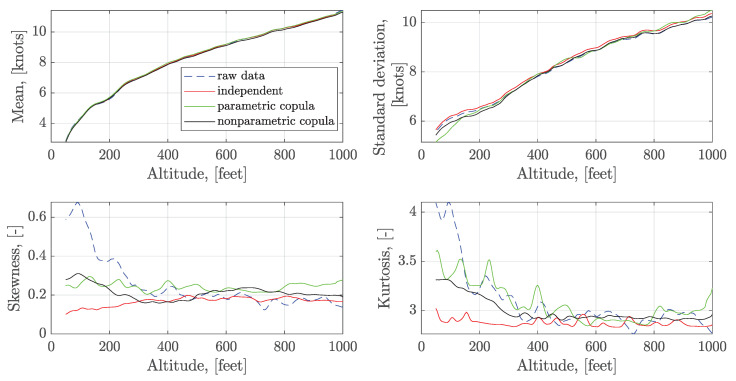
Statistical moments comparison of headwind along with the altitude.

To further analyze the results, the first four order sample moments of the observed wind data along with the altitude are calculated and shown in Figure 9. It shows that the constructed series have a good match at the mean and standard deviation with the raw data. For the independent sampling, the skewness is close to 0, and the kurtosis is always close to 3 for all altitudes. It is close to the moments of Gaussian distribution. In terms of the skewness and kurtosis, results show that the generated series can not exactly capture the skewness and kurtosis. However, the statistical moments using the parametric and nonparametric copula are closer to the raw statistical moments than the independent sampling, especially at the low altitude. Since there is no significant deviation in the numerical value of the skewness and kurtosis, the generated headwind series can be used in simulations for the quantitative assessment.

### 3.3. Windshear

A safety-critical wind series, low-level wind shear, has been recognized as a severe hazard for flight safety. To analyze and quantify the occurrence probability and severity of the wind shear, the significant change of headwind $V_{W}$ with the change of the altitude *h*, so-called horizontal wind shear ramps, are extracted from the flight data. The algorithm is to detect the extreme incremental values of $\Delta V_{W}$ within a certain interval Γ, which is defined as a certain height deviation in this case. A headwind increment for a moving window with a given width of ΔH=Γ/2 is defined as below:

(29) $$\Delta V_{W}=\max V_{W}\left(h:h+\Delta H\right)-V_{W}\left(h\right).$$

**Figure 10:**
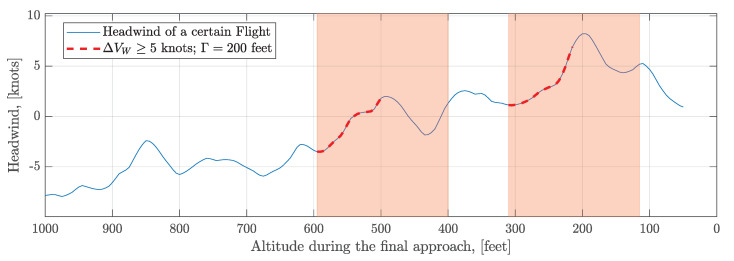
Detection of wind shear ramps in one flight.

A headwind series of one flight is illustrated in Figure 10. The headwind speed incremental threshold $\Delta V_{W}^{*}$ and the detected hight window $\Delta H^{*}$ are predefined. As shown in Figure 10, the incremental threshold $\Delta V_{W}^{*}$ is set to 5 knots (2.57 m/s) and the width of the rolling window ΔH is set to 100 feet. Two wind shear ramps with $\Delta V_{W}\geq 5$ knots and Γ=2ΔH=200 feet are extracted from this flight.

**Figure 11:**
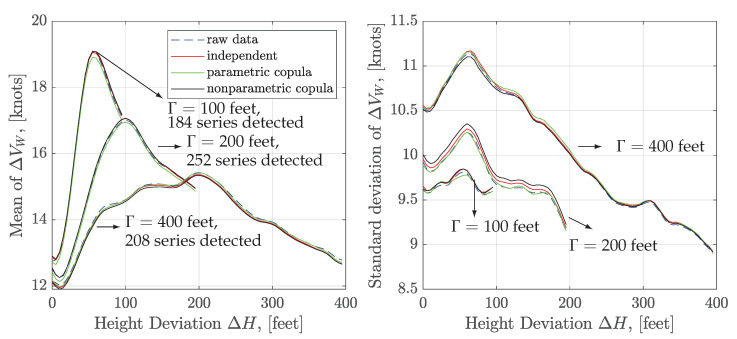
Mean and standard deviation of the extracted and generated wind shear ramps.

The detection algorithm is applied to the 849 flights. The incremental threshold of $\Delta V_{W}$ is set to 5 knots, and the search window is set to 50 feet, 100 feet, and 200 feet, respectively. The mean and standard deviation of the extracted wind shear ramps series and the corresponding number of extracted series are visualized in Figure 11. It shows that the features of the mean and standard deviation depend on the width of the search window.

Given the detected series of wind shear ramps, the KL expansion method allows us to construct new series and enrich the database of the wind shear ramps. The KL truncated order K=10 is used in the construction of wind shear ramps. 5000 new series of $\Delta V_{W}\geq 5$ knots are generated via the independent sampling, parametric and nonparametric copula sampling, respectively. As shown in Figure 11, the mean value of the raw data and generated series match very well, and there are only small biases in the standard deviation. In the case of the Γ=200 feet, the parametric copula sampling has a better match with the raw data than others. The comparison results show that KL constructed model can be used to produce the new wind shear ramps series with similar statistical characteristics.

To further validate if the generated series can be used for the analysis of the wind shear intensity, a metric of $\max\Delta V_{W}/\Delta h$ related to the wind shear intensity is defined using the maximum change of headwind with the altitude as below:

(30) $$\frac{\max\Delta V_{W}}{\Delta h}=\frac{\max V_{W}\left(h\right)-\min V_{W}\left(h\right)}{h_{V_{W,max}}-h_{V_{W,min}}},\;h\in \left[0,\Gamma \right].$$

**Figure 12:**
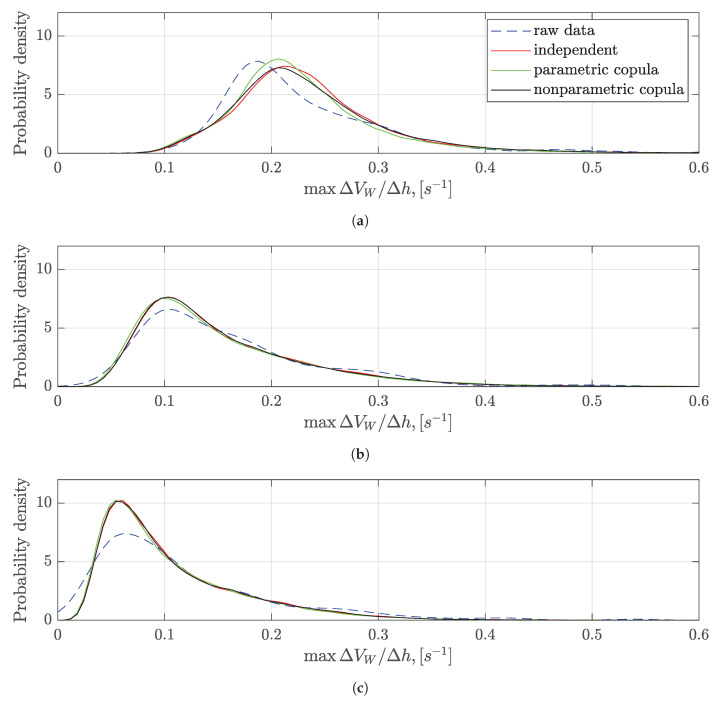
The estimated PDFs of the change rate of headwind along with the altitude. (**a**) The change rate of headwind for $\Delta V_{W}\geq 5$ knots, Γ=100 feet; (**b**) The change rate of headwind for $\Delta V_{W}\geq 5$ knots, Γ=200 feet; (**c**) The change rate of headwind for $\Delta V_{W}\geq 5$ knots, Γ=400 feet.

The metric $\max\Delta V_{W}/\Delta h$ is computed using the raw data and generated series data, respectively. The probability densities of the metric in the three cases are compared. The PDFs of the metrics are estimated using the kernel density functions. As illustrated in Figure 12, the estimated PDFs based on the raw data and generated new series have a good match for the case Γ=200 feet. However, the small deviation exists for the case Γ=100 feet, and Γ=400 feet. Since the numbers of the detected wind shear ramps are limited, increasing the raw data set might improve the results. Nevertheless, the larger difference in PDFs among the three cases can be captured using the generated series. The constructed results also show the same statistical characteristics in wind shear intensity with the raw data. The KL expansion model of wind shear ramps can be used for the analysis of the wind shear intensity.

## 4. Conclusions

This paper presents an algorithm to construct a stochastic wind model based on the operational flight data using the KL expansion method with the vine copula dependence model. The generated wind series based on the KL construction model has the same statistical characteristics as the raw data. For the stationary wind process like turbulence, the similarity of the KL expansion and SR method is pointed out in terms of construction equations, and the construction results of the turbulence reveal that two construction methods coincide. For the non-stationary wind process, KL construction results of headwind and horizontal wind shear ramps validate that the generated series follow the statistical characteristics of the raw data. Therefore the KL expansion model of wind series can be used in simulations for the quantitative analysis.

The vine copula sampling allows us to capture the dependence among the original random coefficients $\xi_{k}$. Series generated via vine copula sampling reduce the deviation from higher order moments of the raw series compared to independent sampling. For further study, more flight data is required to improve accuracy. Additionally, the constructed wind model will be integrated into the incident model simulation settings to quantify the influence of the wind series on operational flight safety.

## Abbreviations

The following abbreviations are used in this manuscript:

KL	Karhunen–Loève
SR	Spectral representation
PSD	Power spectral density

## Appendix A. Three Dimensional Joint Probability Density Function Decomposition

According to the definition of conditional probability, the joint probability density *f* of three random variables $X_{1},X_{2},X_{3}$ can be reconstructed as below:

(A1) $$f\left(x_{1},x_{2},x_{3}\right)=f_{3|12}\left(x_{3}|x_{1},x_{2}\right)f_{2|1}\left(x_{2}|x_{1}\right)f_{1}\left(x_{1}\right).$$

According to the Equation (12), $f_{3|12}\left(x_{3}|x_{1},x_{2}\right)$ and $f_{2|1}\left(x_{2}|x_{1}\right)$ can be written as below:

(A2) $$\begin{array}{cc} f_{2|1}\left(x_{2}|x_{1}\right) & =\frac{f_{12}\left(x_{1},x_{2}\right)}{f_{1}\left(x_{1}\right)}\\ \; & =c_{12}\left(F_{1}\left(x_{1}\right),F_{2}\left(x_{2}\right)\right)f_{2}\left(x_{2}\right),\\ f_{3|12}\left(x_{3}|x_{1},x_{2}\right) & =c_{13;2}\left(F_{1|2}\left(x_{1}|x_{2}\right),F_{3|2}\left(x_{3}|x_{2}\right)\right)f_{3|2}\left(x_{3}|x_{2}\right)\\ \; & =c_{13;2}\left(F_{1|2}\left(x_{1}|x_{2}\right),F_{3|2}\left(x_{3}|x_{2}\right)\right)c_{23}\left(F_{2}\left(x_{2}\right),F_{3}\left(x_{3}\right)\right)f_{3}\left(x_{3}\right), \end{array}$$

where $c_{ij;k}$ is the copula density between the random variables $X_{i}$ and $X_{j}$ for a given $X_{k}$. After substitution of $f_{3|12}\left(x_{3}|x_{1},x_{2}\right)$ and $f_{2|1}\left(x_{2}|x_{1}\right)$ in Equation (A1), the joint probability density *f* is represented by three bivariate copula densities and three marginal probability densities as below:

(A3) $$\begin{array}{cc} f(x_{1},x_{2},x_{3}) & =c_{13;2}\left(F_{1|2}\left(x_{1}|x_{2}\right),F_{3|2}\left(x_{3}|x_{2}\right)\right)\times c_{23}\left(F_{2}\left(x_{2}\right),F_{3}\left(x_{3}\right)\right)\times c_{12}\left(F_{1}\left(x_{1}\right),F_{2}\left(x_{2}\right)\right)\\ \; & \times f_{1}\left(x_{1}\right)\times f_{2}\left(x_{2}\right)\times f_{3}\left(x_{3}\right). \end{array}$$

## Appendix B. Marginal Distributions of ξ_*k*_ in Headwind Modeling

The fitted marginal distributions can be used to generate new realizations of $\xi_{k}$ individually for independent sampling. In addition, to study dependence among several variables using a copula approach, we also have to first find appropriate marginal distributions for each variable involved and then transform it using the fitted marginal distributions to the uniform copula space. This separates the marginal behavior from the underlying dependence structure, which is then captured by the copula.

**Table A1:** Estimated marginal distributions of $\xi_{k}$ in headwind modeling.

$\xi_{k}$	Distribution	Parameters
$\xi_{1}$	Generalized Extreme Value	[k,σ,μ]=[−0.2220,0.9704,−0.3804]
$\xi_{2}$	Logistic	[μ,σ]=[−0.0118,0.5587]
$\xi_{3}$	t Location-Scale	[μ,σ,ν]=[−0.0222,0.8706,8.1359]
$\xi_{4}$	t Location-Scale	[μ,σ,ν]=[−0.0263,0.7279,3.9941]
$\xi_{5}$	t Location-Scale	[μ,σ,ν]=[−0.0158,0.7763,4.8160]
$\xi_{6}$	t Location-Scale	[μ,σ,ν]=[0.0141,0.7007,3.6527]
$\xi_{7}$	t Location-Scale	[μ,σ,ν]=[−0.0501,0.7124,3.7186]
$\xi_{8}$	t Location-Scale	[μ,σ,ν]=[0.0060,0.6046,2.7169]
$\xi_{9}$	t Location-Scale	[μ,σ,ν]=[−0.0234,0.6777,3.3137]
$\xi_{10}$	t Location-Scale	[μ,σ,ν]=[0.0051,0.6529,3.2108]
$\xi_{11}$	t Location-Scale	[μ,σ,ν]=[−0.0087,0.5859,2.4526]
$\xi_{12}$	t Location-Scale	[μ,σ,ν]=[−0.0262,0.6376,2.9137]
$\xi_{13}$	t Location-Scale	[μ,σ,ν]=[0.0075,0.5823,2.4866]
$\xi_{14}$	t Location-Scale	[μ,σ,ν]=[0.0231,0.6695,3.2488]
$\xi_{15}$	t Location-Scale	[μ,σ,ν]=[−0.0345,0.6110,2.6175]
$\xi_{16}$	t Location-Scale	[μ,σ,ν]=[−0.0016,0.6387,2.9972]
$\xi_{17}$	t Location-Scale	[μ,σ,ν]=[−0.0095,0.5603,2.3219]
$\xi_{18}$	t Location-Scale	[μ,σ,ν]=[0.0147,0.5131,2.1901]
$\xi_{19}$	t Location-Scale	[μ,σ,ν]=[−0.0003,0.5795,2.4952]
$\xi_{20}$	t Location-Scale	[μ,σ,ν]=[−0.0211,0.6468,2.9694]

## Appendix C. Dependence of ξ_*k*_ in Headwind Modeling

In Figure A1, marginal histograms of $\xi_{k}^{U}$ are shown in the diagonal. The number from 1 to 20 denotes the corresponding variable $\xi_{k}$. All marginal histograms of $\xi_{k}^{U}$ are close to uniform, which indicates that the estimated marginal distributions are appropriate. The upper triangle shows the sample plots of the copula data $\xi_{k}^{U}$, and the number marked in red color is the estimated Kendall’s τ between two $\xi_{k}^{U}$. Empirical contour plots of the normalized copula data $\xi_{k}^{Z}$ are shown in the lower triangle.

As the truncated order of the KL expansion is set to 20 in the case of headwind construction, 20 dependent random variables $\xi_{k}$ are obtained. Therefore, $\frac{20\cdot (20-1)}{2}=190$ bivariate copula densities are estimated. Normalized contour plots of the first three kinds of conditional copula density are shown in Figure A2. The parametric copula density and nonparametric bivariate copula density functions are used, respectively. The label above the contour plot indicates the related random variables $\xi_{k}$. For example, the label of first top left copula ’2,5;3,15’ indicates $c_{2,5;3,15}$, which is the copula density between the random variables $\xi_{2}$ and $\xi_{5}$ for the given $\xi_{3}$ and $\xi_{15}$. The concentric circles in the contour plots indicate that the two variables are independent, which is obvious in many contour pots of Figure A2b. Otherwise, dependence exists. Several dependence structures are apparent, especially in unconditional bivariate copula densities, as shown in the last row of Figure A2a,b.
